# Anticoccidial activity of natural plants extracts mixture against *Eimeria tenella*: An *in vitro* and *in vivo* study

**DOI:** 10.3389/fvets.2022.1066543

**Published:** 2022-11-23

**Authors:** Mingzheng Han, Wenfeng Hu, Tong Chen, Hanxing Guo, Jianfeng Zhu, Feng Chen

**Affiliations:** ^1^College of Animal Science, South China Agricultural University, Guangzhou, China; ^2^Bioforte Biotechnology (Shenzhen) Co., Ltd., Shenzhen, China; ^3^Research Institute, Wen's Foodstuffs Group Co., Ltd., Yunfu, China; ^4^College of Food Science, South China Agricultural University, Guangzhou, China

**Keywords:** anticoccidial agent, broiler, coccidiosis, *Eimeria tenella*, essential oil, plant extract, poultry

## Abstract

Coccidiosis, an acute epidemic intestinal disease of poultry, is caused by the parasitic protozoan genus *Eimeria*, with *Eimeria tenella* being the most pathogenic spp. Novel approaches are required to address the limitations of current treatments for this disease. We investigated the effects of eight plant extracts and essential oils and their mixture on *Eimeria tenella* as potential treatments for coccidial infection. The anticoccidial effects of non-toxic concentrations of *Punica granatum* L. (0.005 mg/mL), *Plantago asiatica* L. (0.780 mg/mL), *Bidens pilosa* L. (0.390 mg/mL), *Acalypha australis* L. (0.390 mg/mL), *Pteris multifida* Poir (0.050 mg/mL), and *Portulaca oleracea* L. sp. Pl. (0.050 mg/mL) extracts; *Artemisia argyi* Levl. et Vant. (0.010 μL/mL) and *Camellia sinensis* (L.) O. Ktze (0.050 μL/mL) essential oils; and their mixture (0.500 mL/mL) on *Eimeria tenella* were determined using cell viability assays, flow cytometry, and *in vivo* studies. The eight plant extracts and essential oils and their mixture inhibited *Eimeria tenella* sporozoites from invading chicken embryo fibroblast cells *in vitro*. The extract and essential oil mixture improved the feed conversion ratio and body weight gain, reduced fecal oocyst excretion, substantially reduced the mortality of *Eimeria tenella*-infected chickens, and reduced *Eimeria tenella*-induced cecal damage *in vivo*. The results suggest that the extract and essential oil mixtures inhibit *Eimeria tenella* invasion both *in vitro* and *in vivo*, demonstrating their potential as anticoccidial agents.

## Introduction

Coccidiosis is a complex disease caused by parasites belonging to the genus *Eimeria*. Common parasitic spp. of the *Eimeria* genus include *Eimeria acervulina, Eimeria brunetti, Eimeria maxima, Eimeria mitis, Eimeria necatrix, Eimeria praecox*, and *Eimeria tenella* (ET), with ET being the most pathogenic spp. (1). In chickens, infectious ET sporozoites (ETS) enter the cecal mucosa by invading the intestinal mucosal epithelial cells and they then damage the cecal epithelium, which results in reduced feed efficiency and weight gain, bloody stools, and death (2).

After sporulated oocysts enter the host, sporocysts are released owing to mechanical friction in the gizzard. Sporozoites escape from the sporocysts under the influence of bile and trypsin and invade intestinal mucosal epithelial cells, where schizogony and gametogony occur (3). As sporozoite invasion of intestinal mucosal epithelial cells is essential for coccidiosis to occur, it represents one of the main targets for anticoccidial drugs (4). *In vitro* studies on the inhibition of sporozoite invasion of host cells may provide insights to aid in the development of novel strategies to prevent and control coccidiosis. *In vitro* cultures provide a sterile environment for coccidia development, facilitating research on the underlying mechanism(s) of parasite invasion. Several cell lines, such as chick kidney, Madin–Darby bovine kidney (MDBK), and chicken embryo fibroblast (DF-1) cells, have been used to study sporozoite infection and first-generation schizogony. Of these, DF-1 cells are considered a suitable *in vitro* model for studying the inhibition of ET invasion (5, 6).

Primary measures for preventing and controlling coccidiosis in chickens include the use of polyether ionophore antibiotics, chemically synthesized anticoccidial drugs, vaccines, and herbal medicines. However, drug resistance remains a major limitation. Multidrug resistance and cross-resistance reduce the efficacy of anticoccidial drugs (3, 7, 8). The ban on the use of anticoccidial agents as feed additives may be the main motivation for expanding research on plant extracts and essential oils. Although vaccination is an effective alternative to drugs for coccidiosis control, the cumbersome production process, cost of developing and licensing new vaccines, and risk of pathogen dissemination (with attenuated coccidiosis vaccine administration) limit its application (3, 9, 10). Therefore, research on alternative anticoccidial agents, such as plant extracts and essential oils, has gained attention.

Plant extracts and essential oils are relatively producer- and consumer-friendly, environmentally safe, and have been shown to alleviate several ailments. For example, *Punica granatum* L. extract (PGE) is used for deworming, to treat abdominal pain caused by worm infections, and to treat diarrhea and bleeding (11). While *Plantago asiatica* L. extract (PAE) can be used to treat diarrhea, dysentery, and hematuria (blood in the urine) (12), *Bidens pilosa* L. extract (BPE) may help treat enteritis (13), *Acalypha australis* L. extract (AAE) acts as an astringent hemostatic and helps treat enteritis, hematuria, and hematochezia (bloody stools) (14), and *Pteris multifida* Poir extract (PMPE) helps to stop bleeding and can be used to treat enteritis, hematuria, and hematochezia (15). Furthermore, *Portulaca oleracea* L. sp. Pl. extract (POE) can alleviate dysentery and hematochezia (16), *Artemisia argyi* Levl. et Vant. essential oil (AAO) exhibits anti-inflammatory and hemostatic effects (17), and *Camellia sinensis* (L.) O. Ktze essential oil (CSO) has anti-inflammatory and insect repellent properties (18).

Plant extracts and essential oils have low risk of resistance development. Various plant extracts and essential oils exhibit anticoccidial activity by preventing infection or parasite dispersion (19–22). PGE reduces the oocyst output of ET-infected chickens, while increasing the feed conversion ratio (FCR) and average weight, indicating the potential for coccidiosis control (23). PAE reduces weight loss and fecal oocyst excretion in ET-infected chickens (24). BPE reduces fecal oocyst excretion, intestinal pathology, weight loss, and mortality in ET-infected chickens, confirming its therapeutic potential for coccidiosis (25). These findings highlight the potential of plant extracts and essential oils to aid in drug development against coccidiosis. However, to the best of our knowledge, the mechanism(s) underlying the anticoccidial activities of the eight aforementioned plant extracts and essential oils and their mixture have not yet been elucidated.

In this study, we investigated the effects of these eight plant extracts and essential oils and their mixture on ET *in vitro*. The extract and essential oil mixture (EM) was also studied *in vivo* as a potential treatment for coccidiosis. To determine whether an extract or essential oil or the extract and essential oil mixture (EM) inhibited ET infection, we fluorescently labeled ETS *in vitro* and performed invasion inhibition assays to determine the effects of the eight plant extracts and essential oils and their mixture on ETS invasion of DF-1 cells. We also investigated the inhibitory effect of the EM on ET infection *in vivo*. This study lays the foundation for the development of anticoccidial agents in a clinical setting.

## Materials and methods

### Ethics statement

All experimental procedures complied with the ARRIVE guidelines and were in accordance with the recommendations outlined by the Guide for the Care and Use of Laboratory Animals (Ministry of Science and Technology of the People's Republic of China). The research protocol (SYXK-2014-0136) was approved by the Animal Care Committee of South China Agricultural University. All biological safety and sanitation measures were taken.

### ET oocysts, experimental chickens, cells, and extract and essential oil preparation

ET oocysts were provided by Foshan Standard Bio-Tech Co. Ltd. (China). One-day-old, yellow-feathered broilers that were neither vaccinated nor administered drugs were supplied by Wen's Foodstuffs Group Co. Ltd. (China). All biological safety and sanitation measures were taken. Chickens had *ad libitum* access to food until they were 21 days old. DF-1 cells were provided and maintained by the Research Institute of Wen's Foodstuffs Group Co. Ltd. Cells were cultured in Dulbecco's modified eagle medium (DMEM; HyClone, USA) supplemented with 100 U/mL penicillin (Gibco, USA), 100 U/mL streptomycin (Gibco), and 10% fetal bovine serum (FBS; Bovogen, Australia). PGE (Batch No. 20210721), PAE (Batch No. 20210702), BPE (Batch No. 20210629), AAE (Batch No. 20210813), PMPE (Batch No. 20210724), POE (Batch No. 20210712), AAO (Batch No. 210824), and CSO (Batch No. 210809) were purchased from Shaanxi Jiahe Biotechnology Co. Ltd. (China). *In vitro* study, PGE, PAE, BPE, AAE, PMPE, and POE solutions were prepared in DMEM supplemented with 5% FBS, and AAO and CSO solutions were prepared in DMEM supplemented with 5% FBS and 2% Tween-80 (Sangon Biotech Co. Ltd., China), which has no inhibitory effect on ET (26). *In vivo* study, PGE, PAE, BPE, AAE, PMPE, and POE solutions were prepared in ultrapure water, and AAO and CSO solutions were prepared in ultrapure water supplemented 2% Tween-80. The EM was prepared by combining the maximum non-toxic concentrations of the eight aforementioned plant extracts and essential oils for *in vitro* and *in vivo* studies, respectively.

### Cytotoxicity assay

The cytotoxicity of the eight plant extracts and essential oils in DF-1 cells was determined by Cell Counting Kit-8 (CCK-8) assay (Yeasen Biotech, China), following the manufacturer's instructions. After inoculating 5 × 10^4^ DF-1 cells per well in a 96-well plate, the plate was placed in a 5% CO_2_ incubator (Heracell 150i; Thermo Fisher, China) at 37°C for 24 h. Each well was washed three times with 100 μL of 1 × PBS (Thermo Fisher) and incubated with 100 μL of DMEM supplemented with 5% FBS (mock group) or PGE (0.0800–0.0006 mg/mL), PAE (50.00–0.39 mg/mL), BPE (0.780–0.006 mg/mL), AAE (0.780–0.006 mg/mL), PMPE (0.390–0.003 mg/mL), POE (0.780–0.006 mg/mL), AAO (0.390–0.003 μL/mL), or CSO (0.390–0.003 μL/mL) (extract and essential oil groups). Observations were recorded after 24 and 48 h of culture. Each extract and essential oil was evaluated in triplicate by adding CCK-8 (10 μL) and DMEM (90 μL) to each well and incubating at 37°C for 1 h. Absorbance was measured at 450 nm using a microplate reader (Multiskan FC; Thermo Fisher). Cell viability was calculated as follows:


(1) $$\begin{array}{l} Cell\;viability\;\left(\%\right)\;=\;\frac{\left(A_{extract}\;-\;A_{blank}\right)}{\left(A_{mock}\;-\;A_{blank}\right)}\;\times \;100 \end{array}$$

where A = absorbance, extract = extract and essential oil group, mock = mock group, and blank = wells without cells or extracts and essential oils.

The EM was prepared by diluting 2-fold with DMEM supplemented with 5% FBS. EM cytotoxicity (0.008–1.000 mL/mL) was evaluated, following the same procedure used for the individual extracts and essential oils, using CCK-8 to determine the non-toxic concentration of the EM.

### Rejuvenation and passage of oocysts and sporozoite extraction

Yellow-feathered broilers (14-day-old) without coccidial infection were inoculated with 1 × 10^4^ ET oocysts for virulence rejuvenation and passage, as previously described (27). The food and water were free of coccidiosis and anticoccidial drugs. Seven days post-challenge, the chickens were euthanized by cervical dislocation and dissected to collect the cecum and excreta.

Oocysts were collected, purified, sporulated, as previously described (28), and observed under a microscope (DM4 B; Leica, Germany) (29). Interspecific molecular characterization was performed to identify the ET strain (30). Sporulated oocysts were collected 24 h after sporulation, washed three times with 1 × PBS, to prevent any toxic effects, and centrifuged at 4,000 × *g* for 5 min to remove potassium dichromate. Oocysts were sterilized in 30% sodium hypochlorite (Biosharp, China) for 30 min, and centrifuged at 4,000 × *g* for 5 min to remove sodium hypochlorite. Glass beads (diameter: 0.05 mm) (Sigma-Aldrich, USA) were added at an equal volume to the precipitate. Samples were vortexed and observed under a microscope using a hemocytometer (Marienfeld, China). After vortexing, 90% of sporulated oocysts ruptured to release sporocysts, which were collected by centrifugation at 4,000 × *g* for 5 min. Subsequently, 2.5% trypsin (Biosharp) and 10% fresh chicken bile were added, and the samples were incubated at 41°C and 150 rpm for 60 min in an incubator shaker (IS-RDD3; Crystal, USA). The digested samples were observed under a microscope using a hemocytometer. When 90% of sporozoites had been released from the sporocysts, the samples were centrifuged at 4,000 × *g* for 5 min. The sporozoites were resuspended in Hank's balanced salt solution (Thermo Fisher) and filtered through a G3 funnel using a vacuum pump for purification, as previously described (31).

### Fluorescent labeling of sporozoites and invasion rate in DF-1 cells

Extracted sporozoites were fluorescently labeled using a carboxyfluorescein diacetate-succinimidyl ester (CFDA-SE) kit (Beyotime, China), according to the manufacturer's instructions, and detected using an inverted fluorescence microscope (Leica) (31–34). As previously described by Jiang et al. (5, 6), after inoculating 2 × 10^5^ DF-1 cells per well in a 24-well plate, the cells were cultured at 37°C for 24 h in a 5% CO_2_ incubator. The cells were washed with PBS to remove the culture medium. When the cells in each well had formed a monolayer with a density of 90%, they were counted using an automated cell counter (Countess 3 FL; Thermo Fisher). Fluorescently labeled sporozoites were added to the respective wells at a sporozoite:cell ratio of 3:1, and 500 μL of the maximum non-toxic concentration of each extract and essential oil or the EM was added to each well for each extract and essential oil group. The mock group received 500 μL of DMEM supplemented with 5% FBS. The ET group received 500 μL of DMEM supplemented with 5% FBS and fluorescently labeled sporozoites at a sporozoite:cell ratio of 3:1. All groups were evaluated in triplicate. The cells were cultured at 37°C for 12 h in a 5% CO_2_ incubator. The supernatant was removed, and the cells were washed three times with PBS before digestion with 100 μL of 0.25% Trypsin-EDTA (1×) (Gibco, China) for 1 min. Trypsin was removed by centrifugation, and the cells were resuspended in 1 mL of PBS.

A high-speed cell sorter (MoFlo XDP; Beckman Coulter, China) was used to determine the invasion rate of sporozoites infecting DF-1 cells. Standard filter settings were used to measure CFDA-SE-labeled samples using FL-1 and FL-2 detectors at an excitation wavelength of 488 nm (green fluorescence). Summit 5.5 (Beckman Coulter) was used to analyze the fluorescence intensity. All tests were performed in triplicate under identical conditions. The results were visualized using dot plots.

### *In vivo* studies

**Figure 1:**
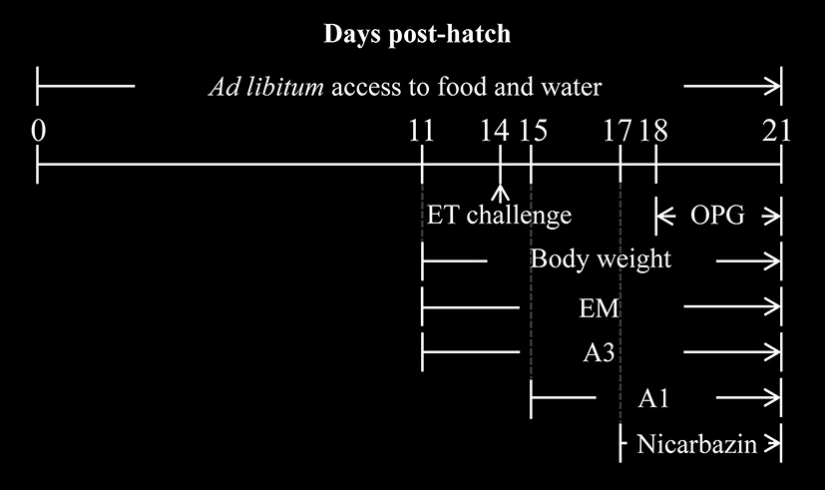
Diagrammatic representation of the *in vivo* studies. The days post hatch of the ET challenge (arrow), drug administration, and body weight measurements are indicated. A3 and A1 represent the EM treatment 3 days before and 1 day after ET infection, respectively. EM, extract and essential oil mixture (non-toxic concentrations of *Punica granatum* L., *Plantago asiatica* L., *Bidens pilosa* L., *Acalypha australis* L., *Pteris multifida* Poir, *Portulaca oleracea* L. sp. Pl. extracts and *Artemisia argyi* Levl. et Vant. and *Camellia sinensis* [L.] O. Ktze essential oils); ET, *Eimeria tenella*; OPG, oocysts per g of excreta.

Yellow-feathered broilers (11 days old; *n* = 360) were randomly divided into six groups (three replicates per group; 20 chickens per replicate). The average weight of the chickens in each group was identical. The groups were named according to the type and timing of extract and essential oil administration: Mock, ET, A3, A1, Nicarbazin, and EM control. The experimental design is shown in Figure 1. The ET group was used as the challenge control in which 4 × 10^4^ ET was inoculated on Day 14 without any other treatment. To simulate preventive treatment in the A3 group, 5 mL of oral EM solution per chicken was administered daily from 3 days pre-challenge until the end of the study (11–21 days old). To simulate therapeutic treatment in the A1 group, 5 mL of oral EM solution per chicken was administered daily from 1-day post-challenge until the end of the study (15–21 days old). In the Nicarbazin group, the anticoccidial Nicarbazin (Hangzhou Fenghe Biotechnology Co. Ltd., China) was administered according to the manufacturer's instructions [400 mg of Nicarbazin was mixed with 1 kg of feed, which was administered from 3 days post-challenge (17–21 days old)]. In the EM control group, 5 mL of oral EM solution per chicken was administered daily from Day 11 to Day 21, to determine its effect on chickens not exposed to any other treatment. In the mock group, 5 mL of 1 × PBS was administered daily from Day 11 to Day 21 as the non-challenge control in which chickens received food and water without anyother treatment. Each group of chickens was raised in a separate cage with sufficient space. All chickens had *ad libitum* access to food and water and were not administered any anticoccidial drugs or vaccines.

### Evaluation of the FCR, relative body weight gain, survival rate, oocysts per g of excreta index, cecal lesion score index, and anticoccidial index *in vivo*

The food intake, body weight, and number of dead chickens in each group were recorded daily post-challenge. The FCR, RBWG, and SR were calculated as follows:


(2) $$\begin{array}{l} FCR\;\left(\%\right)\;=\;\frac{Average\;daily\;food\;intake\;per\;group}{Average\;daily\;weight\;gain\;per\;group}\;\times \;100 \end{array}$$

(3) $$\begin{array}{l} RBWG\;\left(\%\right)=\;\frac{Body\;weight\;gain\;per\;group}{Body\;weight\;gain\;of\;the\;mock\;group}\;\times \;100 \end{array}$$

(4) $$\begin{array}{l} SR\;\left(\%\right)=\frac{Total\;number\;of\;chickens\;per\;group\;-\;The\;number\;of\;dead\;chickens\;per\;group}{Total\;number\;of\;chickens\;per\;group\;}\\ \;\times \;100 \end{array}$$

The excreta of each group were collected and weighed on Days 4–7 p.i. (18–21 days old) to calculate the OPG index, following the standard protocol (35, 36). Briefly, oocyst suspensions were prepared by diluting 1 g of excreta from each group with distilled water, followed by serial filtration using WS Tyler sieves (pore sizes: 1 mm, 250 μm, and 45 μm). After centrifugation, oocysts were suspended in a saturated salt solution and mixed thoroughly. The homogeneous suspension was transferred to two McMaster chambers to obtain oocyst counts (three technical replicates per sample). The OPG was obtained using the average of each sample, and the OPG index was calculated as follows:


(5) $$\begin{array}{l} OPG\;index\;=\;\frac{Oocyst\;ratio}{g\;of\;excreta}\;\times \;100\;\times \;0.4 \end{array}$$

where the oocyst ratio per g of excreta = ([OPG per group]/[OPG of the ET group]) × 100.

On Day 7 p.i. (21 days old), the chickens were dissected, and cecal lesions were examined to determine the cecal LSI. The cecum was collected, as previously described (37), preserved in 4% paraformaldehyde, and outsourced to Wuhan Saiweier Biological Technology Co. Ltd. (China) for pathological tissue sectioning. The macroscopic damage caused by ET to the cecum was graded from 0 to 4, as follows: 0, normal tissue with no visible damage; 1, scattered spotted hemorrhage on the cecal wall with typical intestinal wall and content; 2, slightly thickened intestinal wall with a small amount of mixed-blood cecal content and numerous bleeding lesions; 3, cecal core with blood coagulation due to extensive cecal bleeding and a deformed or atrophied cecum with a thickened cecal wall; and 4, lesions extending to the rectum, severe cecal atrophy, and coagulated cecal core with a thickened cecal wall (38). If the extent of cecal lesions on both sides was inconsistent, the side with more severe lesions was used to grade the macroscopic damage caused by ET to the cecum. Chickens that died from coccidiosis were scored as 4. The cecal LSI was calculated as follows:


(6) $$\begin{array}{l} LSI\;=\;Lesion\;score\;per\;group\;\times \;10 \end{array}$$

Finally, the anti-ET effect of the EM was determined using the ACI, which was calculated as follows as previously described (38, 39):


(7) $$\begin{array}{l} ACI\;=\;RBWG\;+\;SR\;-\;\left(OPG\;index\;+\;LSI\right) \end{array}$$

where an ACI < 120, 120–160, and ≥ 160 indicates resistance, partial resistance, and sensitivity to the anticoccidial agent, respectively.

### Statistical analyses

Data are expressed as means ± SD. SPSS Statistics (version 22; IBM Corp., USA) was used for all statistical analyses. One-way ANOVA and Tukey's test were used to determine significant differences between groups. Multivariate analysis of variance was performed to determine the significant differences in FCR between groups on Days 1-7 p.I. Differences between groups were significant at *P* < 0.05. Figures were generated using GraphPad Prism 7.04 (GraphPad Software, USA) and Summit 5.5 (Beckman Coulter).

## Results

### Evaluation of cytotoxicity

**Figure 2:**
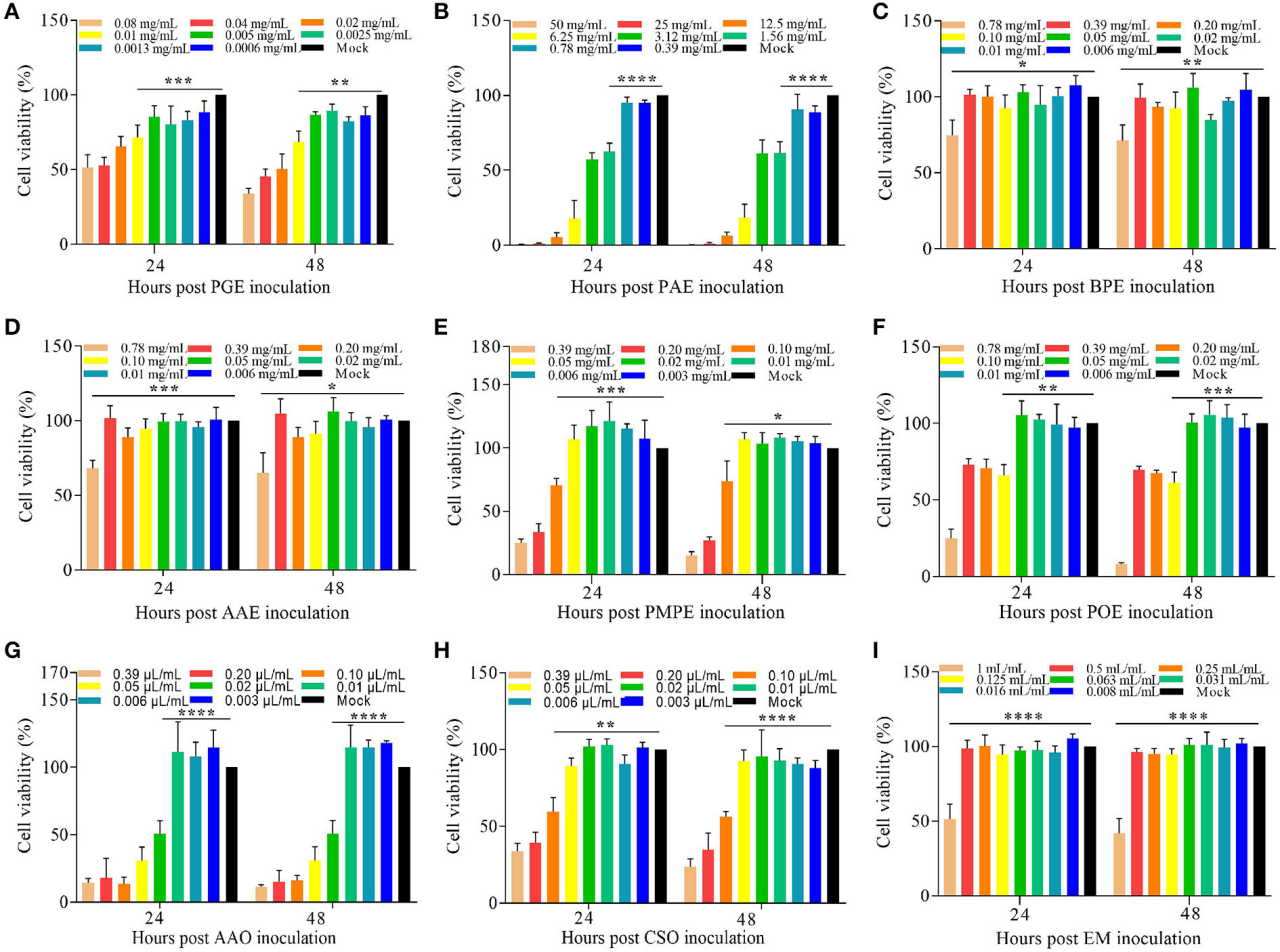
Cytotoxicity of the eight plant extracts and essential oils and the EM in DF-1 cells *in vitro*. Viability (%) of DF-1 cells cultured with various concentrations of **(A)** PGE, **(B)** PAE, **(C)** BPE, **(D)** AAE, **(E)** PMPE, **(F)** POE, **(G)** AAO, **(H)** CSO, and **(I)** the EM relative to the Mock 24 and 48 h before the CCK-8 assay. Data are expressed as means ± S.D. of three independent experiments. *P < 0.05, **P < 0.01, ***P < 0.005, ****P < 0.001. AAE, *Acalypha australis* L. extract; AAO, *Artemisia argyi* Levl. et Vant. essential oil; BPE, *Bidens pilosa* L. extract; CCK-8, Cell Counting Kit-8; CSO, *Camellia sinensis* (L.) O. Ktze essential oil; DF-1, chicken embryo fibroblast; EM, extract and essential oil mixture (non-toxic concentrations of PGE, PAE, BPE, AAE, PMPE, POE, AAO, and CSO); PAE, *Plantago asiatica* L. extract; PGE, *Punica granatum* L. extract; PMPE, *Pteris multifida* Poir extract; POE, *Portulaca oleracea* L. sp. Pl. extract.

The relative viability of DF-1 cells was evaluated after the administration of different concentrations of plant extracts and essential oils. The non-toxic concentrations of PGE, PAE, BPE, AAE, PMPE, POE, AAO, CSO, and the EM were 0.005 mg/mL, 0.780 mg/mL, 0.390 mg/mL, 0.390 mg/mL, 0.050 mg/mL, 0.050 mg/mL, 0.010 μL/mL, 0.050 μL/mL, and 0.50 mL/mL, respectively (Figures 2A–I). The non-toxic concentrations of these eight plant extracts and essential oils and the EM were used to determine their effects on ETS invasion *in vitro*.

### Plant extracts and essential oils inhibited ETS invasion *in vitro*

**Figure 3:**
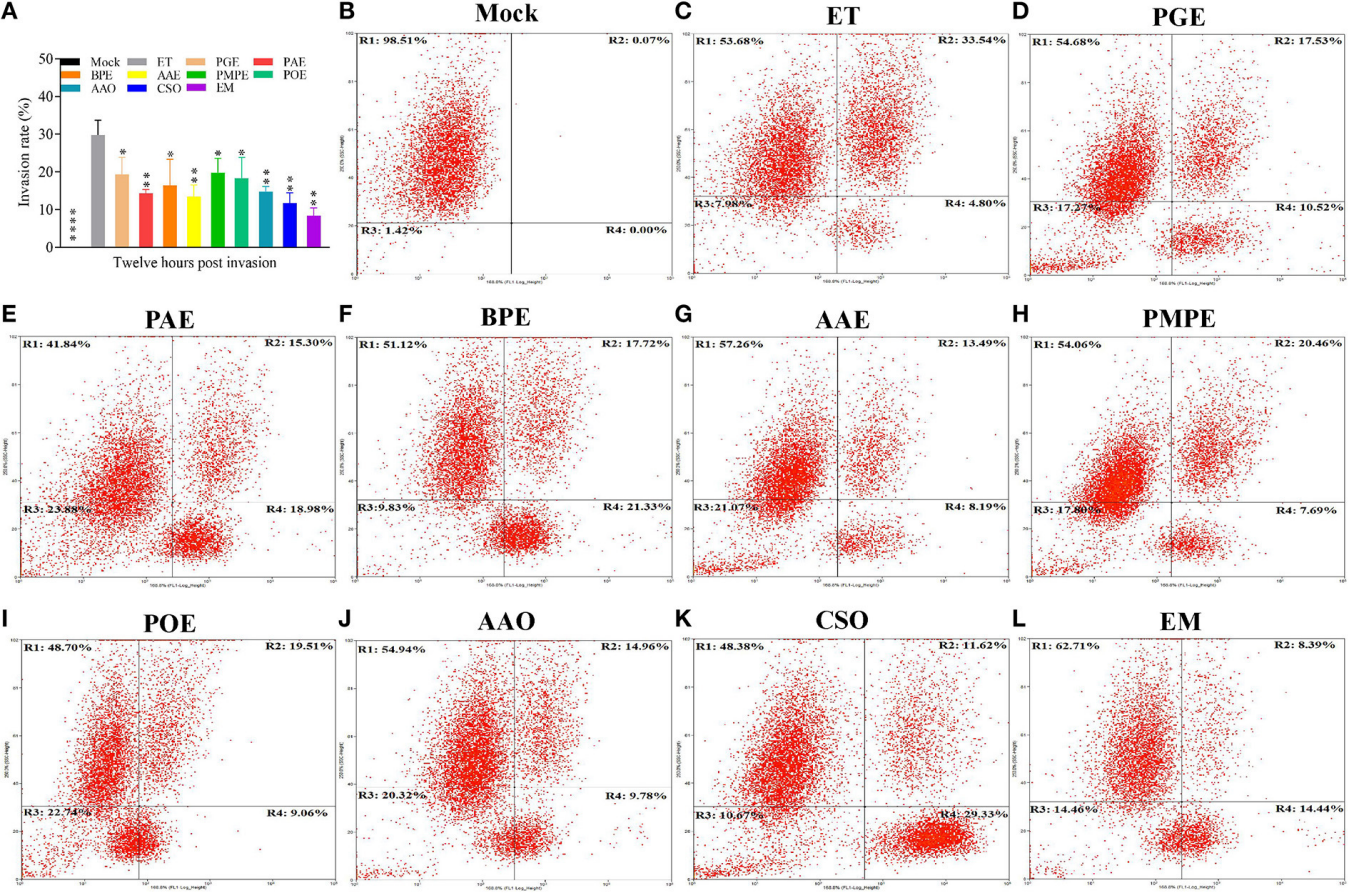
Invasion inhibition of the eight plant extracts and essential oils and the EM *in vitro*. Flow cytometry was performed 12 h after sporozoites were cultured with the individual extracts and essential oils, EM, or mock in DMEM. **(A)** Invasion rate (%) of the ET and samples administered to one of the eight plant extracts and essential oils or the EM. Results represent three independent experiments. *P < 0.05, **P < 0.01, ****P < 0.001 (each group compared with the ET group). Representative plots of the **(B)** mock, **(C)** ET, **(D)** PGE, **(E)** PAE, **(F)** BPE, **(G)** AAE, **(H)** PMPE, **(I)** POE, **(J)** AAO, **(K)** CSO, and **(L)** EM control groups. R1, ETS uninfected cells; R2, ETS-infected cells; R3, cell fragments; R4, ETS of uninfected cells. AAE, *Acalypha australis* L. extract; AAO, *Artemisia argyi* Levl. et Vant. essential oil; BPE, *Bidens pilosa* L. extract; CSO, *Camellia sinensis* (L.) O. Ktze essential oil; EM, extract and essential oil mixture (non-toxic concentrations of PGE, PAE, BPE, AAE, PMPE, POE, AAO, and CSO); ET, *Eimeria tenella*; ETS, ET sporozoites; PAE, *Plantago asiatica* L. extract; PGE, *Punica granatum* L. extract; PMPE, *Pteris multifida* Poir extract; POE, *Portulaca oleracea* L. sp. Pl. extract.

Flow cytometry revealed that invasion rate was highest in ET group (ETS cultured without treatment) and was substantially reduced in ETS cultured with PGE, PAE, BPE, AAE, PMPE, POE, AAO, CSO, and the EM compared to ET group (*P* < 0.05, *P* < 0.01, *P* < 0.05, *P* < 0.01, *P* < 0.05, *P* < 0.05, *P* < 0.01, *P* < 0.01, and *P* < 0.01, respectively; Figures 3A–L), demonstrating the inhibitory effects of the eight plant extracts and essential oils and the EM on ETS invasion of DF-1 cells. ETS invasion was lowest in the EM group and was, therefore, selected for further anti-ET analysis *in vivo*. Different methods of administration were used to explore the anti-ET effect of the EM. Furthermore, a Nicarbazin group was set up to compare the effects of anticoccidial agent and EM and an EM control group was used to determine its adverse effects on the host.

### Effects of the EM on the chicken FCR and RBWG post-challenge

**Figure 4:**
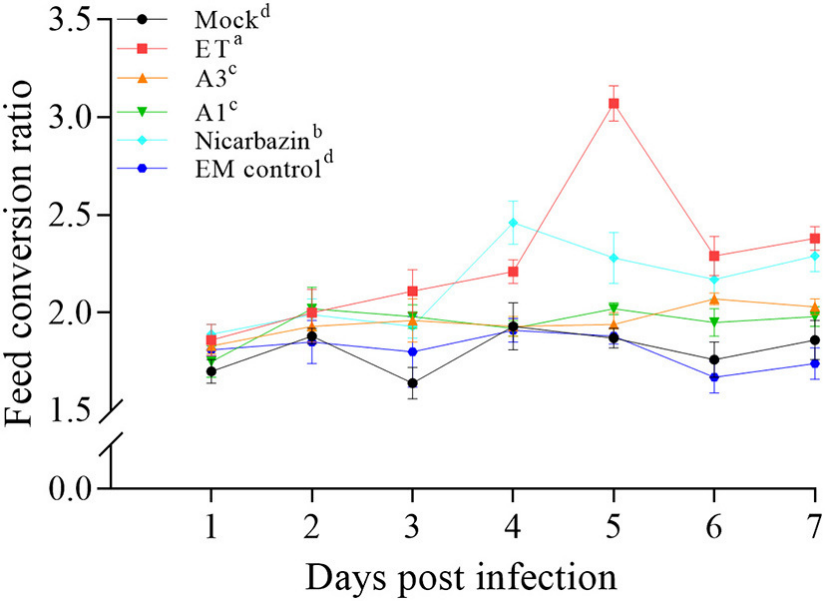
Evaluation of the FCR of chickens *in vivo* from Day 1–7 p.i. A3 and A1 represent the EM treatment 3 days before and 1 day after ET infection, respectively. EM, extract and essential oil mixture (non-toxic concentrations of *Punica granatum* L., *Plantago asiatica* L., *Bidens pilosa* L., *Acalypha australis* L., *Pteris multifida* Poir, and *Portulaca oleracea* L. sp. Pl. extracts and *Artemisia argyi* Levl. et Vant. and *Camellia sinensis* [L.] O. Ktze essential oils); ET, *Eimeria tenella*; FCR, feed conversion ratio. Lowercase letters (a-d) indicate statistically significant differences (*P* < 0.05).

As weight gain is affected by the ET infection, we examined the effects of the EM on weight gain in ET-infected chickens. Before treatment, we ensured that the weight of each group was consistent to avoid errors caused by individual differences. We compared the FCRs by recording each group's food intake and the average daily weight gain. The FCR of the EM control groups was not significantly different from that of the mock group (Figure 4), suggesting that EM supplementation had no adverse effects on the feeding and growth of chickens. Besides, the FCR of the A3, A1, and Nicarbazin groups was significantly lower than that of the ET group (*P* < 0.001, *P* < 0.001, and *P* < 0.05, respectively; Figure 4), indicating that the EM and Nicarbazin reduced the FCR of ET-infected chickens.

**Figure 5:**
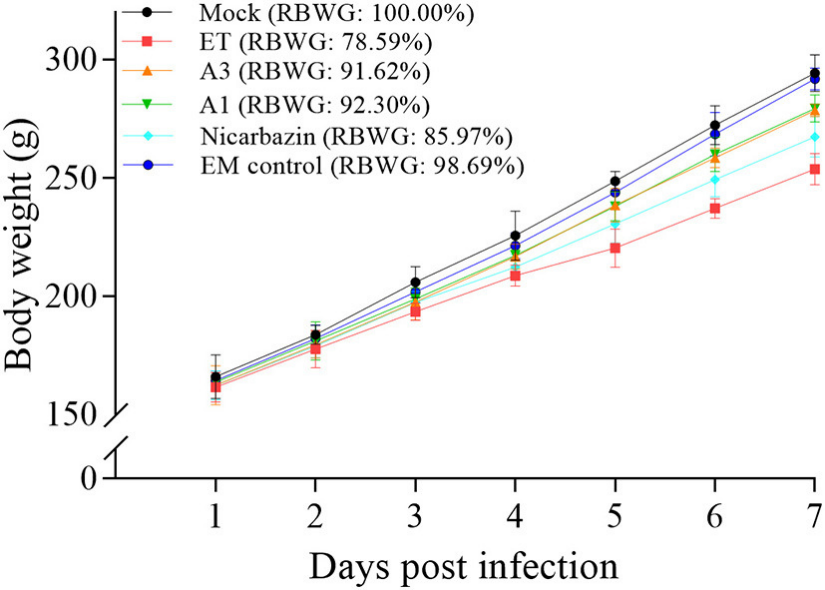
Evaluation of chicken RBWG *in vivo* from Day 1–7 p.i. A3 and A1 represent EM treatment 3 days before and 1 day after ET infection, respectively. EM, extract and essential oil mixture (non-toxic concentrations of *Punica granatum* L., *Plantago asiatica* L., *Bidens pilosa* L., *Acalypha australis* L., *Pteris multifida* Poir, and *Portulaca oleracea* L. sp. Pl. extracts and *Artemisia argyi* Levl. et Vant. and *Camellia sinensis* [L.] O. Ktze essential oils); ET, *Eimeria tenella*; RBWG, relative body weight gain.

To better understand the differences in weight, we calculated the RBWG of each group relative to the mock group. The RBWG of the A3, A1, and Nicarbazin groups was higher than that of the ET group and was similar to that of the mock group (Figure 5), indicating that the EM improved the RBWG of ET-infected chickens when used as a preventive or therapeutic measure. Moreover, the RBWG of the EM control group was similar to that of the mock group, indicating that the EM had no significant adverse effects on chickens.

### Effects of the EM on the chicken SR post-challenge

**Figure 6:**
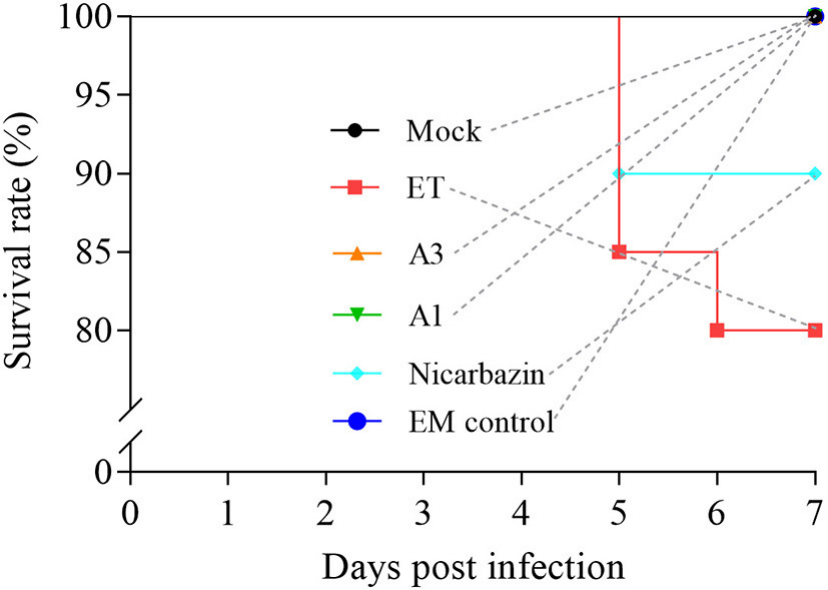
Evaluation of the chicken SR *in vivo* from Day 1–7 p.i. A3 and A1 represent EM treatment 3 days before and 1 day after ET infection, respectively. EM, extract and essential oil mixture (non-toxic concentrations of *Punica granatum* L., *Plantago asiatica* L., *Bidens pilosa* L., *Acalypha australis* L., *Pteris multifida* Poir, and *Portulaca oleracea* L. sp. Pl. extracts and *Artemisia argyi* Levl. et Vant. and *Camellia sinensis* [L.] O. Ktze essential oils); ET, *Eimeria tenella*.

The high mortality rates that result from ET infection are of great concern, and their reduction is an important treatment goal. We recorded the SR of ET-infected chickens post-challenge to determine the effects of the EM on the SR. The SR was 100% in the A3 and A1 groups and 90% in the Nicarbazin group, which were both higher than that in the ET group (80%) (Figure 6), indicating that the EM reduced mortality and exerted a protective effect on the ET-infected chickens.

### Effect of the EM on chicken oocyst excretion post-challenge

**Figure 7:**
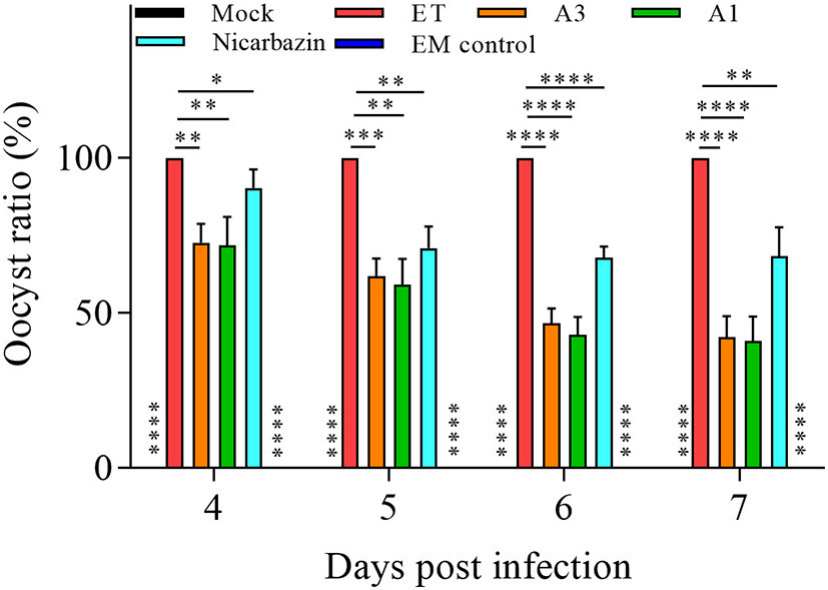
Evaluation of the oocyst ratio per g of excreta from the chickens *in vivo* from Day 4–7 p.i. *P < 0.05,**P < 0.01, ***P < 0.005, ****P < 0.001 (each group compared with the ET group). A3 and A1 represent EM treatment 3 days before and 1 day after ET infection, respectively. EM, extract and essential oil mixture (non-toxic concentrations of *Punica granatum* L., *Plantago asiatica* L., *Bidens pilosa* L., *Acalypha australis* L., *Pteris multifida* Poir, and *Portulaca oleracea* L. sp. Pl. extracts and *Artemisia argyi* Levl. et Vant. and *Camellia sinensis* [L.] O. Ktze essential oils); ET, *Eimeria tenella*.

To further explore the anticoccidial effect of the EM *in vivo*, fecal oocysts (an indicator of ET propagation and transmission) were counted in chickens post-challenge (35, 36). From days 4–7 p.i. (18–21 days old), the oocyst ratio per g of excreta was significantly lower in the A3, A1, and Nicarbazin groups than in the ET group (*P* < 0.01, *P* < 0.005, *P* < 0.001, and *P* < 0.001; and *P* < 0.01, *P* < 0.01, *P* < 0.001, and *P* < 0.001; and *P* < 0.05, *P* < 0.01, *P* < 0.001, and *P* < 0.01, respectively; Figure 7), indicating that the EM reduced oocyst excretion in ET-infected chickens.

### Effects of the EM on chicken cecal lesions post-challenge

**Figure 8:**
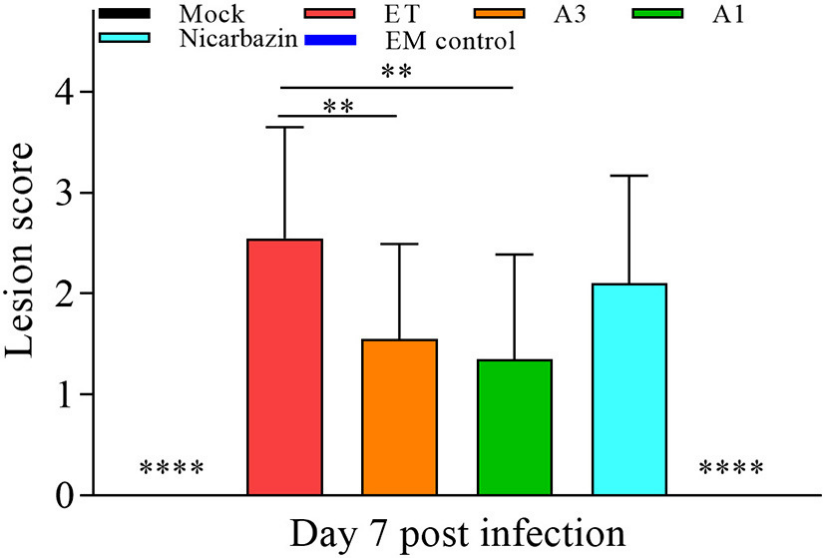
Evaluation of chicken cecal lesion scores *in vivo* on Day 7 p.i. **P < 0.01, ****P < 0.001 (each group compared with the ET group). A3 and A1 represent EM treatment 3 days before and 1 day after ET infection, respectively. EM, extract and essential oil mixture (non-toxic concentration of *Punica granatum* L., *Plantago asiatica* L., *Bidens pilosa* L., *Acalypha australis* L., *Pteris multifida* Poir, and *Portulaca oleracea* L. sp. Pl. extracts and *Artemisia argyi* Levl. et Vant. and *Camellia sinensis* [L.] O. Ktze essential oils); ET, *Eimeria tenella*.

As ET invades and damages the cecum, it is critical that the EM has a protective effect. Cecal lesions were blindly scored according to traditional evaluation criteria (37), to objectively determine the effect of the EM on cecal lesions (37). The cecal lesion score of the A3 and A1 groups was significantly lower than that of the ET group (both *P* < 0.01; Figure 8), indicating that the EM alleviated ET-induced cecal lesions. There was no significant difference between the ET and Nicarbazin groups, indicating that the effect of Nicarbazin on ET infection may reduced.

### Effects of the EM on the chicken cecum post-challenge

**Figure 9:**
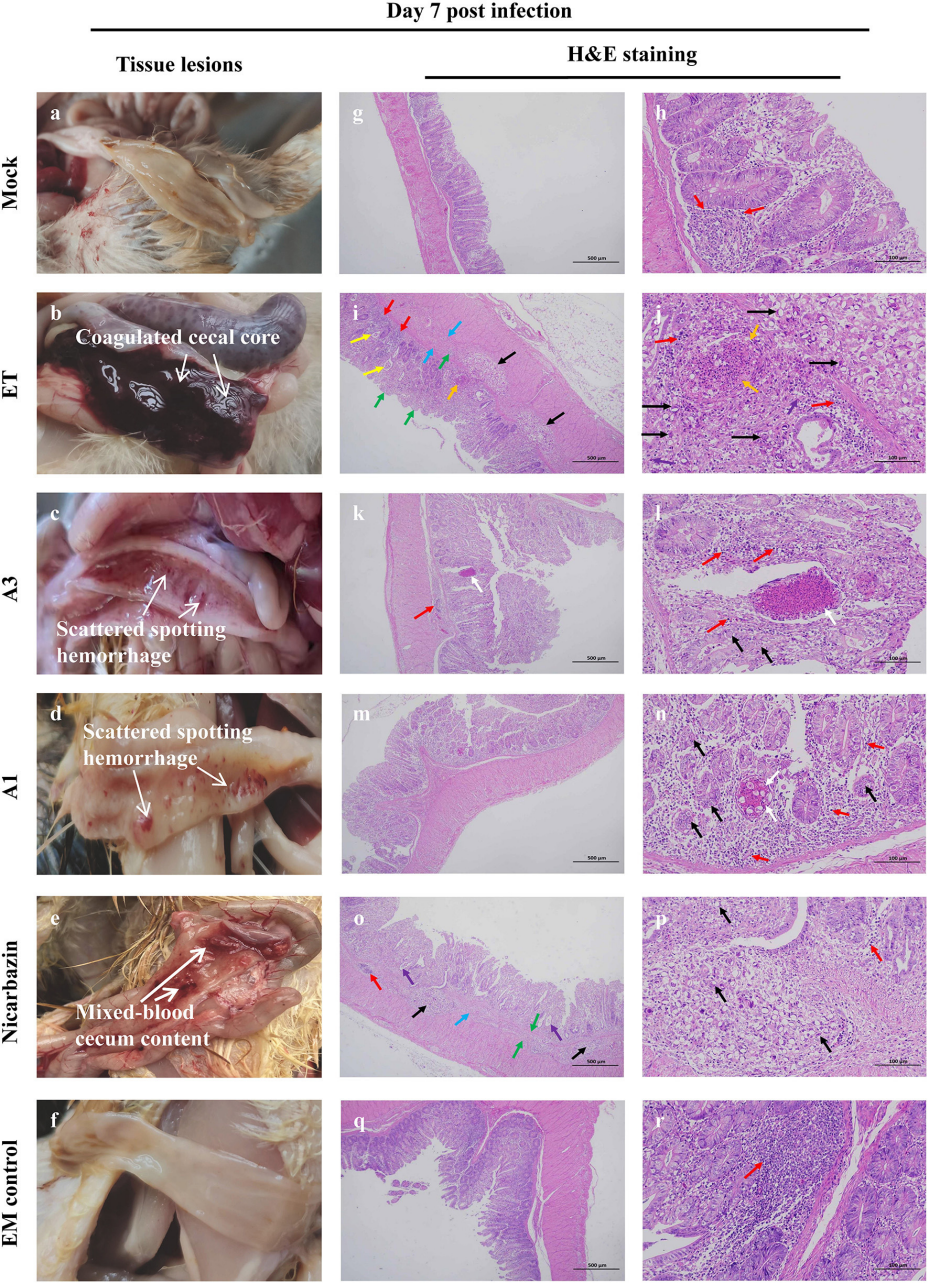
Macroscopic lesions and histopathology of the cecum from chickens administered EM on Day 7 p.i. Macroscopic cecal lesions in the **(a)** mock, **(b)** ET, **(c)** A3, **(d)** A1, **(e)** nicarbazin, and **(f)** EM control groups. Arrows indicate characteristic lesions. Histopathological sections (two panels for each group [representing the same sample at different scales]) of the cecum in the **(g,h)** mock, **(i,j)** ET, **(k,l)** A3, **(m,n)** A1, **(o,p)** nicarbazin, and **(q,r)** EM control groups analyzed on Day 7 p.i. Arrows indicate lymphoid tissue infiltration (red), oocysts (black), connective tissue proliferation (green), oocyst proliferation in the glandular cavity (yellow), heterophilic granulocyte infiltration (purple), submucosal damage and inflammatory cell infiltration (blue), necrotic tissue and cell fragments (orange), and eosinophilic enhancement (white). A3 and A1 represent EM treatment 3 days before and 1 day after ET infection, respectively. EM, extract and essential oil mixture (non-toxic concentrations of *Punica granatum* L., *Plantago asiatica* L., *Bidens pilosa* L., *Acalypha australis* L., *Pteris multifida* Poir, and *Portulaca oleracea* L. sp. Pl. extracts and *Artemisia argyi* Levl. et Vant. and *Camellia sinensis* [L.] O. Ktze essential oils); ET, *Eimeria tenella*.

Autopsy results of 21-day-old chickens (day 7 p.i.) (Figures 9a–f) showed that a cecal core formed because of extensive blood coagulation and intestinal wall thickening (Grade 3) in the ET group, whereas only scattered spotted hemorrhage (Grade 1) was observed in the A3 and A1 groups. Furthermore, bleeding lesions accompanied by mixed-blood cecum contents appeared (i.e., grade 2) in the Nicarbazin group. Observations in the EM control group were similar to those in the mock group; no pathological features were observed.

Microscopic lesions were also examined. There was negligible diffuse lymphatic tissue infiltration in the lamina propria in the mock group (Figures 9g,h); no parasites were observed. The mucosa and submucosa indicated several aggregated or scattered oocysts in the ET group (Figures 9i,j). A reduction in the size of the enteraden and connective tissue hyperplasia were observed. Several intestinal glands were dilated, and oocysts were observed in the glandular lumen. Lymphoid tissue and slight heterophilic infiltration were observed in the mucosa. Extensive damage to the submucosa was observed, including submucosal connective tissue proliferation, gap widening, inflammatory cell infiltration, and ulceration. Small tissue necrosis and cell fragmentation were observed in the mucosa. In the A3 group, oocysts were observed in the intestinal glandular epithelium with greater lymphatic tissue infiltration in the lamina propria and submucosa (Figures 9k,l). Several small necrotic foci and eosinophilic enhancements were observed in the mucosa. In the A1 group, oocysts were observed in the intestinal glandular epithelium (Figures 9m,n). The lamina propria and submucosa were infiltrated by scattered lymphoid tissue with occasional small necrotic foci, cell fragments, and increased eosinophilia. In the Nicarbazin group (Figures 9o,p), the submucosa had many oocysts, which were aggregated or scattered. In addition, reduced intestinal glands, submucosal space widening, connective tissue proliferation, and forming ulcerations were observed. A few intestinal glands were dilated, and oocysts could be seen in the glandular lumen. Minimal infiltration of lymphoid tissue in the mucosa and submucosa was observed. Furthermore, abundant intestinal mucosal glands and slight lymphatic tissue infiltration in the lamina propria were observed in the EM control group (Figures 9q,r).

### Evaluation of ET sensitivity to the chicken EM

The sensitivity of ET to the EM was measured using the ACI (an indicator of *Coccidioides*) (38, 39). The ACI of each group was calculated using the RBWG, SR, cecal LSI, and number of OPG (Table 1). The ACI of the A3, A1, and Nicarbazin groups were both higher than 120. As the ACI was approximately 160 in the A3 and A1 groups, ET was considered sensitive to the EM, indicating that the EM protected chickens against ET infection *in vivo*.

**Table 1:** Anticoccidial activity of the plant extract and essential oil mixture p.i.

**Group (*n =* 20)**	**SR^a^ (%)**	**RBWG^a^ (%)**	**OPG index^a^**	**Cecal LSI^a^**	**ACI index^a^**
Mock^b^	100.00	100.00	0.00	0.00	200.00
ET^c^	80.00	78.59	40.00	25.50	93.09
A3^d^	100.00	91.62	16.91	15.50	159.21
A1^d^	100.00	92.30	16.42	13.50	162.38
Nicarbazin	90.00	85.97	27.34	21.00	127.63
EM control	100.00	98.69	0.00	0.00	198.69

a SR, RBWG, OPG index, cecal LSI, and ACI were calculated using the formulae described in the Materials and methods.

b Mock represents the non-challenge control in which chickens received food and water without any treatment.

c ET represents the ET challenge control in which chickens were inoculated with ET without any treatment.

d A3 and A1 represent EM treatment 3 days before and 1 day after ET infection, respectively.

ACI, anticoccidial index; EM, extract and essential oil mixture (non-toxic concentrations of *Punica granatum* L., *Plantago asiatica* L., *Bidens pilosa* L., *Acalypha australis* L., *Pteris multifida* Poir, and *Portulaca oleracea* L. Sp. Pl. extracts and *Artemisia argyi* Levl. et Vant. and *Camellia sinensis* [L.] O. Ktze essential oils); ET, *Eimeria tenella*; LSI, lesion score index; OPG, oocysts per g of excreta; RBWG, relative body weight gain; SR, survival rate.

## Discussion

Coccidial prevention and control is a major priority for the poultry industry, wherein plant extracts and essential oils have emerged as potential anticoccidial agents (40, 41). Traditionally, coccidiostat screening is performed *in vivo*, where its advantages and disadvantages are determined based on the SR, RBWG, OPG index, and cecal LSI, making it time-consuming. The primary focus of coccidiosis research is to develop less time- and effort-consuming techniques to identify anticoccidial or coccidiostatic agents. With the development of cell culture technology for *Coccidioides*, the evaluation of anticoccidial activity can now be performed in cellular systems. This time-saving development enables preliminary screening of anticoccidial drugs *in vitro*, followed by *in vivo* studies for further validation. Thus, *in vitro* screening is expected to facilitate research, albeit with certain limitations. To date, chicken coccidiosis cell culture research has focused primarily on ET and *Eimeria acervulina*, and less on the other five *Eimeria* spp. (*Eimeria brunetti, Eimeria maxima, Eimeria mitis, Eimeria necatrix*, and *Eimeria praecox*), limiting coccidiosis research at the cellular level. Two culture systems are available for cytological research on ET: primary and passage cell cultures. Considering the challenges associated with the preparation, culture, and life cycle of primary cells, MDBK and DF-1 cells are used as passage cell lines for *in vitro* studies (5, 42). However, more coccidial cell culture systems are needed to guide future research.

Establishing a reliable detection method based on objective measurements of sporozoite invasion is crucial for studying anticoccidial agents at the cellular level. Bumstead and Tomley (43) stained coccidia-infected cells with H&E and measured the invasion rate based on microscopic examination of coccidia-containing vacuoles. This method was subjective and, therefore, limited. To circumvent this, a fluorescent probe, CFDA-SE (which binds irreversibly to ETS proteins after stable and uniform labeling) (32, 44), was used to label ETS in this study. The invasion rate was calculated more accurately using flow cytometry. CFDA-SE fluorescent markers and flow cytometry revealed that the eight plant extracts and essential oils and the EM inhibited ETS invasion of DF-1 cells, suggesting that the eight plant extracts and essential oils and the EM play an inhibitory role in ETS invasion. Coccidia belong to the Apicomplexa. Apicomplexan invasion of host cells can be divided into three stages: gliding motility, moving junction, and parasitophorous vacuole (45, 46). However, understanding at what stage the eight plant extracts and essential oils and the EM affect invasion requires further investigation. Baba et al. (47) showed that, during invasion, D-galactose residues on the surface of ETS recognize host cell surface receptors, which may be associated with parasite invasion. Furthermore, del Cacho et al. (48) showed that ETS lipid rafts mediate their invasion of host cells. For example, flotillin-1, a lipid raft molecular tag protein, plays a pivotal role in ETS invasion and participates in cell invagination. Bussière et al. (49) reported that the mitogen-activated protein kinase signaling pathway also plays a pivotal role in ETS invasion. However, whether the mechanism(s) of inhibition of the eight plant extracts and essential oils and the EM are related to the aforementioned mechanisms of ETS invasion requires further investigation.

It is also important to explore the synergistic and antagonistic effects of the eight plant extracts and essential oils and the EM. Our results showed that when the eight plant extracts and essential oils were mixed, the inhibitory effect of the EM on ETS invasion was more pronounced. Previous studies have shown that PGE, PAE, AAE, PMPE, POE, and AAO have hemostatic or therapeutic effects on hematochezia (11, 12, 14–17), and BPE, AAE, PMPE, AAO, and CSO have therapeutic or anti-inflammatory effects on enteritis (13–15, 17, 18). Therefore, studies exploring whether mixing the extracts and essential oils could have a synergistic effect, which better explains the observation that the EM more strongly inhibits ETS invasion than the individual extracts and essential oils, are needed. Our findings reveal insights into the mechanism of action of the eight plant extracts and essential oils and the EM, laying the foundation for further research on the mechanism(s) of the inhibition of ETS invasion.

ET infection causes extensive cecal injury, poor growth performance, and high mortality *in vivo* (2). By comparing the histopathology and cecal lesion scores of each group *in vivo*, we demonstrated that the EM reduced oocyst excretion, mitigated damage to the intestinal mucosa caused by cleavage, and reduced ulcer formation and connective tissue proliferation, alleviating ET-induced cecal injury. The EM reduced mortality and the FCR, increased body weight, and decreased oocyst excretion in ET-infected chickens.

Drug resistance against almost all anticoccidial drugs has been reported (50), which is a major factor in controlling coccidiosis. Our findings showed that the anti-coccidial effect of Nicarbazin on ET slightly decrease, indicating that ET may develop resistance to Nicarbazin, which may relevant to the widespread use of Nicarbazin. This observation is consistent with the drug resistance problem associated with anti-coccidiosis drugs (51–55). The ACI showed that ET resistance to the EM was low, indicating that the EM had an inhibitory effect on ET infection. We identified the EM as a potential agent that could reduce the economic losses caused by ET infection and control its spread in ET-contaminated farms. Moreover, the following questions remain unanswered: Which combination of these eight plant extracts and essential oils provides the maximum inhibition to ET infection? Can a superior alternative to the EM be developed? What are the critical components of the plants extracts and EM that confer inhibition to ET? Does the EM confer protection against other similar parasites, such as *Eimeria acervulina, Eimeria maxima*, and *Eimeria necatrix*? Addressing these questions will help reduce the incidence of coccidiosis and allow a better understanding of the mechanism(s) of resistance to ET infection.

## Conclusion

To the best of our knowledge, this is the first study to investigate the invasion-inhibitory effects of eight plant extracts and essential oils, both alone and in combination, at the cellular level. Our results suggest that the EM has anticoccidial activity against ET *in vitro* and *in vivo*. The EM may thus be a potential anticoccidial agent for the prevention and control of ET infection in the future.

## Data availability statement

The original contributions presented in the study are included in the article/supplementary material, further inquiries can be directed to the corresponding author/s.

## Ethics statement

The animal study was reviewed and approved by the research protocol (SYXK-2014-0136) and the Animal Care Committee of South China Agricultural University.

## Author contributions

MH: conceptualisation, methodology, formal analysis, investigation, resources, data curation, writing–original draft, writing–review and editing, vizualisation, and project administration. WH: investigation and resources. TC and JZ: project administration. FC: writing–review and editing, supervision, and project administration. All authors read and approved the final manuscript.

## Funding

This work was supported by the Provincial Science and Technology Special Fund of Yunfu, Guangdong Province, China [Grant No. 2019A090202]. The funding source had no role in the study design; the collection, analysis, or interpretation of data; the writing of the report; or the decision to submit the article for publication.

## Conflict of interest

All authors are affiliated with Bioforte Biotechnology (Shenzhen) Co., Ltd., and the Research Institute, Wen's Foodstuffs Group Co., Ltd.

## Publisher's note

All claims expressed in this article are solely those of the authors and do not necessarily represent those of their affiliated organizations, or those of the publisher, the editors and the reviewers. Any product that may be evaluated in this article, or claim that may be made by its manufacturer, is not guaranteed or endorsed by the publisher.

## References

[1] Chapman HD Shirley MW. The Houghton strain of *Eimeria tenella*: a review of the type strain selected for genome sequencing. Avian Pathol. (2003) 32:115–27. 10.1080/0307945021000071588 12745365

[2] Alkhudhayri AA Dkhil MA Al-Quraishy S. Nanoselenium prevents eimeriosis-induced inflammation and regulates mucin gene expression in mice jejunum. Int J Nanomedicine. (2018) 13:1993–2003. 10.2147/IJN.S162355 29662312 PMC5892949

[3] Blake DP Tomley FM. Securing poultry production from the ever-present *Eimeria* challenge. Trends Parasitol. (2014) 30:12–9. 10.1016/j.pt.2013.10.003 24238797

[4] Augustine PC. Cell: sporozoite interactions and invasion by apicomplexan parasites of the genus *Eimeria*. Int J Parasitol. (2001) 31:1–8. 10.1016/S0020-7519(00)00150-8 11286188

[5] Jiang LL Lin JJ Han HY Dong H Zhao QP Zhu SH. Establishment and application of DF-1 cell culture system for the sporozoites of *Eimeria tenella*. Chin Veter Sci. (2011) 41:551–6. 10.16656/j.issn.1673-4696.2011.06.010 [Article in Chinese]

[6] Jiang L Lin J Han H Dong H Zhao Q Zhu S. Identification and characterization of *Eimeria tenella* apical membrane antigen-1 (AMA1). PLoS ONE. (2012) 7:e41115. 10.1371/journal.pone.0041115 22829917 PMC3400601

[7] Clarke L Fodey TL Crooks SR Moloney M O'Mahony J Delahaut P. A review of coccidiostats and the analysis of their residues in meat and other food. Meat Sci. (2014) 97:358–74. 10.1016/j.meatsci.2014.01.004 24534603

[8] Clark EL Tomley FM Blake DPA. Are *Eimeria* genetically diverse, and does it matter? Trends Parasitol. (2017) 33:231–41. 10.1016/j.pt.2016.08.007 27593338

[9] Witcombe DM Smith NC. Strategies for anti-coccidial prophylaxis. Parasitology. (2014) 141:1379–89. 10.1017/S0031182014000195 24534138

[10] Khater H F Ziam H Abbas A Abbas RZ Raza MA Hussain K. Avian coccidiosis: Recent advances in alternative control strategies and vaccine development. Agrobiol Rec. (2020) 1:11–25. 10.47278/journal.abr/2020.00315839405

[11] Ge S Duo L Wang J Yang J Li Z Tu Y. A unique understanding of traditional medicine of pomegranate, Punica granatum L and its current research status. J Ethnopharmacol. (2021) 271:113877. 10.1016/j.jep.2021.113877 33515685

[12] Haddadian K Zahmatkash M. A review of *Plantago* plant. Indian J Trad Knowl. (2014) 13:681–5. Available online at: http://nopr.niscpr.res.in/handle/123456789/29518

[13] Abiodun OO Sosanya AS Nwadike N Oshinloye AO. Beneficial effect of *Bidens pilosa* L. (Asteraceae) in a rat model of colitis. J Basic Clin Physiol Pharmacol. (2020) 31:166. 10.1515/jbcpp-2019-0166 32598311

[14] Palaniswamy UR. Taiwanese native medicinal plants: phyto-pharmacology and therapeutic values. HortScience. (2007) 42:183c−4. 10.21273/HORTSCI.42.1.183c

[15] Harinantenaina L Matsunami K Otsuka H. Chemical and biologically active constituents of *Pteris multifida*. J Nat Med. (2008) 62:452–5. 10.1007/s11418-008-0265-9 18553125

[16] Khanam DB Begum W Tipo FA. Pharmacological profile, phytoconstituents, and traditional uses of Khurfa (*Portulaca oleracea L*): Unani perspective. J Pharm Innov. (2019) 8:367–72. Available online at: https://www.thepharmajournal.com/archives/2019/vol8issue4/PartG/8-4-71-811.pdf

[17] Ge YB Wang ZG Xiong Y Huang XJ Mei ZN Hong ZG. Anti-inflammatory and blood stasis activities of essential oil extracted from *Artemisia argyi* leaf in animals. J Nat Med. (2016) 70:531–8. 10.1007/s11418-016-0972-6 26894818

[18] Liu M Wang J Tian B Zefeng X. Proliferation, accumulation of polyphenols, and antioxidant activities of callus from the ‘Anji Baicha' cultivar of tea [Camellia sinensis (L) O Ktze]. Korean J Hortic Sci Technol. (2017) 35:252–64. 10.12972/kjhst.20170028

[19] Quiroz-Castañeda RE Dantán-González E. Control of avian coccidiosis: future and present natural alternatives. BioMed Res Int. (2015) 2015:430610. 10.1155/2015/430610 25785269 PMC4346696

[20] Habibi H Ghahtan N Tohidi S Zarrinfar A. Effect of composition of medicinal plants on growth performance, gut bacteria, hematological parameters, anticoccidial index, and optimum anticoccidial activity in domestic chicken. Comp Clin Path. (2022) 31:737–45. 10.1007/s00580-022-03352-2

[21] Hussain K Abbas RZ Abbas A Samiullah K Ahmed T Siddique F. Anticoccidial potential of Ageratum conyzoides and its effect on Blood parameters of experimentally infected Broiler Chickens. J Hellenic Veter Med Soc. (2021) 72:3085–90. 10.12681/jhvms.28497

[22] Ishaq AN Sani D Abdullahi SA Jolayemi KO Ebbo AA Jatau ID. Evaluation of Anticoccidial Activity of Citrus aurantium L Ethanolic Leaf Extract against Experimental Eimeria tenella Infection in Broiler Chickens (*Gallus gallus domesticus*). Pharmacol Res Mod Chin Med. (2022) 4:100138. 10.1016/j.prmcm.2022.100138

[23] Ahad S Tanveer S Malik TA Nawchoo IA. Anticoccidial activity of fruit peel of *Punica granatum* L. Microb Pathog. (2018) 116:78–83. 10.1016/j.micpath.2018.01.015 29339307

[24] Hong S Oh GW Kang WG Kim O. Anticoccidial effects of the *Plantago asiatica* extract on experimental *Eimeria tenella* infection. Lab Anim Res. (2016) 32:65–9. 10.5625/lar.2016.32.1.65 27051444 PMC4816998

[25] Yang WC Tien YJ Chung CY Chen YC Chiou WH Hsu SY. Effect of *Bidens pilosa* on infection and drug resistance of *Eimeria* in chickens. Res Vet Sci. (2015) 98:74–81. 10.1016/j.rvsc.2014.11.002 25440995

[26] Shetshak MA Jatau ID Suleiman MM Ameh MP Gabriel A Akefe IO. In vitro anticoccidial activities of the extract and fractions of Garcinia kola (Heckel h) against *Eimeria tenella* oocyst. Recent Pat Biotechnol. (2021) 15:76–84. 10.2174/1872208315666210129095213 33511943

[27] Shirley MW. Eimeria species and strains of chickens. In: *Guidelines on Techniques in Coccidiosis Research*. (1995) p. 1–25.

[28] Tomley F. Techniques for isolation and characterization of apical organelles from *Eimeria tenella* sporozoites. Methods. (1997) 13:171–6. 10.1006/meth.1997.0509 9405200

[29] Joyner LP Long PL. The specific characters of the *Eimeria*, with special reference to the coccidia of the fowl. Avian Pathol. (1974) 3:145–57. 10.1080/03079457409353827 18777269

[30] Blake DP Qin Z Cai J Smith AL. Development and validation of real-time polymerase chain reaction assays specific to four species of *Eimeria*. Avian Pathol. (2008) 37:89–94. 10.1080/03079450701802248 18202955

[31] Hermosilla C Stamm I Taubert A Lutz K Zahner H Menge C. Fluorescent *Eimeria bovis* sporozoites and meront stages *in vitro*: a helpful tool to study parasite-host cell interactions. Parasitol Res. (2008) 102:777–86. 10.1007/s00436-007-0849-4 18175149

[32] Jahn D Matros A Bakulina AY Tiedemann J Schubert U Giersberg M. Model structure of the immunodominant surface antigen of *Eimeria tenella* identified as a target for sporozoite-neutralizing monoclonal antibody. Parasitol Res. (2009) 105:655–68. 10.1007/s00436-009-1437-6 19387686

[33] Lyons AB. Analysing cell division *in vivo* and *in vitro* using flow cytometric measurement of CFSE dye dilution. J Immunol Methods. (2000) 243:147–54. 10.1016/S0022-1759(00)00231-3 10986412

[34] Urbani S Caporale R Lombardini L Bosi A Saccardi R. Use of CFDA-SE for evaluating the *in vitro* proliferation pattern of human mesenchymal stem cells. Cytotherapy. (2006) 8:243–53. 10.1080/14653240600735834 16793733

[35] Conway DP Dayton AD Mckenzie ME. Comparative testing of anticoccidials in broiler chickens: the role of coccidial lesion scores. Poult Sci. (1999) 78:529–35. 10.1093/ps/78.4.529 10230905

[36] Haug A Williams RB Larsen S. Counting coccidial oocysts in chicken faeces: a comparative study of a standard McMaster technique and a new rapid method. Vet Parasitol. (2006) 136:233–42. 10.1016/j.vetpar.2005.11.024 16388903

[37] Johnson J Reid WM. Anticoccidial drugs: lesion scoring techniques in battery and floor-pen experiments with chickens. Exp Parasitol. (1970) 28:30–6. 10.1016/0014-4894(70)90063-9 5459870

[38] Li GQ Kanu S Xiang FY Xiao SM Zhang L Chen HW. Isolation and selection of ionophore-tolerant *Eimeria* precocious lines: *E. tenella, E. maxima, and E. Acervulina*. Vet Parasitol. (2004) 119:261–76. 10.1016/j.vetpar.2003.12.009 15154593

[39] Wang Z Shen J Suo X Zhao S Cao X. Experimentally induced monensin-resistant *Eimeria tenella* and membrane fluidity of sporozoites. Vet Parasitol. (2006) 138:186–93. 10.1016/j.vetpar.2006.01.056 16524664

[40] Remmal A Achahbar S Bouddine L Chami N Chami F. *In vitro* destruction of *Eimeria* oocysts by essential oils. Vet Parasitol. (2011) 182:121–6. 10.1016/j.vetpar.2011.06.002 21726944

[41] Orengo J Buendía AJ Ruiz-Ibáñez MR Madrid J Del Río L Catalá-Gregori P. Evaluating the efficacy of cinnamaldehyde and *Echinacea purpurea* plant extract in broilers against *Eimeria acervulina*. Vet Parasitol. (2012) 185:158–63. 10.1016/j.vetpar.2011.09.024 21996002

[42] Patton WH. *Eimeria tenella*: cultivation of the asexual stages in cultured animal cells. Science. (1965) 150:767–9. 10.1126/science.150.3697.767 5844081

[43] Bumstead J Tomley F. Induction of secretion and surface capping of microneme proteins in *Eimeria tenella*. Mol Biochem Parasitol. (2000) 110:311–21. 10.1016/S0166-6851(00)00280-2 11071285

[44] Labbé M De Venevelles P Girard-Misguich F Bourdieu C Guillaume A Pery P. *Eimeria tenella* microneme protein EtMIC3: identification, localisation and role in host cell infection. Mol Biochem Parasitol. (2005) 140:43–53. 10.1016/j.molbiopara.2004.12.002 15694485

[45] Bradley PJ Sibley LD. Rhoptries: an arsenal of secreted virulence factors. Curr Opin Microbiol. (2007) 10:582–87. 10.1016/j.mib.2007.09.013 17997128 PMC2682365

[46] Proellocks NI Coppel RL Waller KL. Dissecting the apicomplexan rhoptry neck proteins. Trends Parasitol. (2010) 26:297–304. 10.1016/j.pt.2010.02.012 20347614

[47] Baba E Uno H Sadano N Fukata T Sasai K Arakawa A. *Eimeria tenella*: Role of carbohydrates on sporozoite at the penetration into cultured cells. Exp Parasitol. (1996) 83:67–72. 10.1006/expr.1996.0050 8654553

[48] del Cacho E Gallego M Sánchez-Acedo C Lillehoj HS. Expression of flotillin-1 on *Eimeria tenella* sporozoites and its role in host cell invasion. J Parasitol. (2007) 93:328–32. 10.1645/GE-992R.1 17539416

[49] Bussière FI Brossier F Le Vern Y Niepceron A Silvestre A de Sablet T. Reduced parasite motility and micronemal protein secretion by a p38 MAPK inhibitor leads to a severe impairment of cell invasion by the apicomplexan parasite *Eimeria tenella*. PLoS ONE. (2015) 10:e0116509. 10.1371/journal.pone.0116509 25689363 PMC4331428

[50] Li GQ Kanu S Xiao SM Xiang FY. Responses of chickens vaccinated with a live attenuated multi-valent ionophore-tolerant *Eimeria* vaccine. Vet Parasitol. (2005) 129:179–86. 10.1016/j.vetpar.2004.09.034 15845272

[51] Chen ZY Zheng MX Gu SP. Sensitivity of the precocious strain of ETenella to several kinds of coccidiostats. Prog Vet Med. (2009) 30:26–9.

[52] Lan LH Sun BB Zuo BX Chen XQ Du AF. Prevalence and drug resistance of avian *Eimeria* species in broiler chicken farms of Zhejiang Province, China. Poult Sci. (2017) 96:2104–9. 10.3382/ps/pew499 28339722

[53] Peek HW Landman WJ. Resistance to anticoccidial drugs of Dutch avian *Eimeria spp*. Field isolates originating from 1996, 1999 and 2001. Avian Pathol. (2003) 32:391–401. 10.1080/0307945031000121149 17585463

[54] Wang F Zeng MH Wu JF Wang JJ. Determination of the different effects of four anticoccidia drugs against field strains of *Eimeria tenella*. J Anhui Agric Univ. (2010) 37:631–4. 10.13610/j.cnki.1672-352x.2010.04.033 [Article in Chinese]

[55] Zhang XZ Zhang YY Jia QH. Studies on drug resistance of *Eimeria tenella* Qinhuangdao strain to seven anticoccidial drugs. J Northwest A & F Univ. (2008) 7:14–8. 10.13207/j.cnki.jnwafu.2008.07.030 [Article in Chinese]

